# A case study of salivary microbiome in smokers and non-smokers in Hungary: analysis by shotgun metagenome sequencing

**DOI:** 10.1080/20002297.2020.1773067

**Published:** 2020-06-07

**Authors:** Roland Wirth, Gergely Maróti, Róbert Mihók, Donát Simon-Fiala, Márk Antal, Bernadett Pap, Anett Demcsák, Janos Minarovits, Kornél L. Kovács

**Affiliations:** a Department of Biotechnology, University of Szeged, Szeged, Hungary; b Institute of Plant Biology, Biological Research Center, Szeged, Hungary; c Department of Operative and Esthetic Dentistry, Faculty of Dentistry, University of Szeged, Szeged, Hungary; d Department of Oral Biology and Experimental Dental Research, Faculty of Dentistry, University of Szeged, Szeged, Hungary

**Keywords:** Smoking, saliva metagenome, read-based taxonomy, genome-centric binning, *Prevotella*, *Megasphaera*, oral cancer

## Abstract

**Objective:**

To investigate the role of cigarette smoking in disease-development through altering the composition of the oral microbial community. Periodontitis and oral cancer are highly prevalent in Hungary; therefore, the salivary microbiome of smoker and non-smoker Hungarian adults was characterized.

**Methods:**

Shotgun metagenome sequencing of salivary DNA samples from 22 individuals (11 non-smokers and 11 current smokers) was performed using the Ion Torrent PGM^TM^ platform. Quality-filtered reads were analysed by both alignment-based sequence similarity searches and genome-centric binning.

**Results:**

*Prevotella, Veillonella* and *Streptococcus* were the predominant genera in the saliva of both groups. Although the overall composition and diversity of the microbiota were similar, *Prevotella* was significantly more abundant in salivary samples of current smokers compared to non-smokers. Members of the genus *Prevotella* were implicated in the development of inflammatory diseases and oral cancer. The abundance of the genus *Megasphaera* also increased in current smokers, whereas the genera *Neisseria, Oribacterium, Capnocytophaga* and *Porphyromonas* were significantly reduced. The data generated by read-based taxonomic classification and genome-centric binning mutually validated the two distinct metagenomic approaches.

**Conclusion:**

Smoking-associated dysbiosis of the salivary microbiome in current cigarette smokers, especially increased abundance of *Prevotella* and *Megasphaera* genera, may facilitate disease development.

## Introduction

The oral cavity of healthy humans harbors a diverse microbial community called the “normal flora”, which is composed of more than 700 bacterial species that regularly attach to and form biofilms on the surfaces of soft and hard tissues within the mouth [1–3]. Members of the oral biofilms are regularly shed into the saliva, which is bathing the oral mucosa [4]. Saliva is a complex biological fluid whose composition is affected both by local conditions in the oral cavity and systemic diseases [5–7]. Since saliva can be collected in a painless, non-invasive manner, substantial efforts have been made to identify disease-related salivary biomarkers, recently [8]. In addition to the biomolecules accumulating in saliva during pathological processes, the oral microbiome may also be regarded as a new biomarker reservoir [9]. Thus, the changes of the salivary microbial community can also be exploited for the diagnosis and monitoring of oral and systemic diseases [10–18]. Smoking is an important risk factor for oral diseases, such as periodontitis and oral cancer, and it is also associated with a wide variety of systemic diseases [19–21]. Although tobacco use, especially cigarette smoking, decreased in the last decades in Western countries, regular smoking is still a common habit in Central- and Eastern European countries [22,23], Asia, China, and North Africa (https://www.who.int/gho/tobacco/use/en/). Tobacco smoke may contain more than 5,000 chemicals, among them toxic, mutagenic and carcinogenic substances [24]. These chemicals may initiate pathogenic alterations by interacting directly with various host cells and extracellular matrix components [25]. Nicotine, a major, highly addictive constituent of cigarette smoke modulates the immune responses [25,26]. Toxic compounds in tobacco smoke may cause cellular injury and cell death whereas carcinogens, including N-nitrosamines and polycyclic aromatic hydrocarbons may initiate tumorigenesis by forming DNA adducts and blocking DNA repair [27–31]. Chemicals in cigarette smoke may also contribute to disease development indirectly by changing the composition of the human oral microbiome [32]. Alteration of the oral microbiome in cigarette smokers may favour disease development by increasing the local density of bacterial pathogens or decreasing the prevalence of their competitors [12,33,34].

Cigarette smoking is one of the most important aspects in the development of oral diseases, including periodontitis and oral cancer, which are particularly prevalent in Hungary [35–37]. Smoking may influence disease progress by altering the microbial communities of the oral cavity; therefore,e in this cross-sectional study, we characterized the salivary microbiome of smoker and non-smoker Hungarian adults. We applied a metagenomic approach and used both alignment-based sequence similarity searches and genome-centric binning for the analysis of shotgun sequences generated by the Ion Torrent PGM^TM^ platform [38–41].

## Study design and recruitment of participants

The study protocol was approved by the Institutional Review Board of the University of Szeged, Szeged, Hungary. Signed informed consent was obtained from each healthy adult participant enrolled into the study at the Department of Operative and Esthetic Dentistry, Faculty of Dentistry, University of Szeged, Hungary. Study participants were divided into two groups, non-smokers and current smokers, based on the data they provided regarding tobacco consumption (cigarette smoking). The smoking exposure of current smokers was calculated in pack-years. One pack contained 20 cigarettes.

## Characteristics of study participants

A total of 11 healthy adult non-smokers (4 males and 7 females; mean age: 40 years; range: 26–46 years), including 8 never smokers and 11 healthy adult current smokers (8 males and 3 females; mean age: 41.5 years; range: 34–61 years) participated in this cross-sectional study. Three of the participants in the non-smoker group quit smoking 5.5, 5 and 1.5 years before the study, respectively. None of the participants suffered from known chronic illness and none were treated with antibiotics at least 6 months prior to sampling. In order to record the oral parameters, all patients received a full mouth cariological and periodontal examination, performed by an experienced practitioner. The number of missing teeth (excluding third molars), Plaque Index (PI; also known as the Silness-Löe Index), bleeding on probing (BOP; the presence or absence of bleeding within 15 sec after probing), probing depth (PD; in millimeters), and clinical attachment level (CAL; to describe the position of the soft tissue in relation to the cemento-enamel junction) were recorded. To describe the periodontal status of the patients, a classification was used [42], which was proven to be reliable in our earlier works [43,44]. All patients with moderate or severe peridontitis were excluded from the study.

## Methods

### Measurement of exhaled carbon monoxide in healthy smokers and non-smokers

The level of exhaled carbon monoxide (CO) is a suitable indicator of smoking status [45,46]. We used a calibrated, portable CO monitor (piCO + Smokerlyzer, Breath CO monitor, Bedfont Scientific Ltd., Kent, UK) to assess the exhaled CO levels in the study groups of non-smokers and current smokers.

Participants were asked to exhale completely, inhale fully, and then hold their breath for as long as possible. Right after this, the participants were instructed to exhale slowly into the unit and exhale fully. This procedure was repeated three times and the mean value was calculated.

### Saliva collection and DNA isolation from saliva

Unstimulated whole saliva samples were collected from the participants by the simple drooling method, aliquoted and stored at −80 C°. After thawing, saliva samples (3 ml, each) were centrifuged at 13 000 rpm for 5 min. DNA extractions were carried out by using the Macherey-Nagel NucleoSpin Soil DNA kit (Macherey-Nagel: 740,780.250). The lysis mixture contained 700 µL SL1 and 150 µL Enhancer SX lysis solutions. After lysis (bead beating), the kit protocol was followed. The quantity of DNA was determined in a NanoDrop ND-1000 spectrophotometer (NanoDrop Technologies, Wilmington, USA) and a Qubit 2.0 Fluorometer (Life Technologies, Carlsbad, USA). DNA purity was tested by agarose gel electrophoresis and on an Agilent 2200 TapeStation instrument (Agilent Technologies, Santa Clara, USA).

### Next-generationn sequencing and bioinformatics analysis

**Figure 1.**
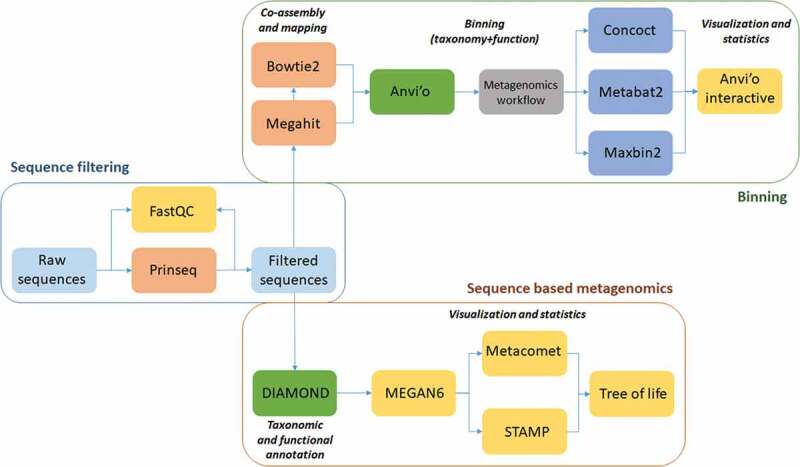
Summary of the data analysis workflow and the employed software packages. The main steps of the initial data filtering and bioinformatics steps to extract the read-based and genome-based metagenome data are boxed separately.

The recommendations of the Ion Torrent PGM™ sequencing platform were closely followed (Life Technologies, Thermo Fisher Scientific, USA). The sample libraries were prepared by Ion Xpress Plus Fragment Library Kit (Cat. No. 4471269; Thermo Fisher Scientific, USA) and quantified by Ion Library TaqMan® Quantitation Kit (Cat. No. 4468802; Thermo Fisher Scientific, USA) with the help of StepOne Real-Time PCR System (Applied Biosystems). Emulsion PCR was performed with OneTouch 2 and Ion OneTouch ES devices by using the Ion PGM Template OT2 200 kit (Cat. No. 4,480,974; Thermo Fisher Scientific, USA). Barcoding was made by Ion Xpress Barcode Adapters 1–16 Kit (Cat. No. 4,471,250¸Thermo Fisher Scientific, USA). Sequencing was performed with Ion PGM 200 Sequencing kit (Cat. No. 4,474,004¸ Thermo Fisher Scientific, USA) on Ion Torrent PGM 316 chip. The raw data have been made publicly available at SRA accession: PRJNA553326 (Release date: 2019–07-09). The workflow of the subsequent data analysis is summarized in Figure 1.

### Raw sequence filtering

Galaxy Europe server was employed to pre-process the raw sequences (www.usegalaxy.eu). Low-quality reads were filtered by Prinseq (min. length: 150 bp; min. score: 15; quality score threshold to trim positions: 20; sliding window used to calculate quality score: 1) [47]. Quality of raw and filtered sequences were checked with FastQC program.

### Read-based metagenomics

Filtered high or moderate quality sequences were further analyzed by Diamond software, applying the LCA (Lowest Common Ancestor) algorithm [48]. Diamond parameters were set as follows: Blast Mode: BlastX, Reference database: NCBI nr database. MEGAN6 was used to add taxon names to Diamond sequence classifications (Min Score: 80, Min support percent: 80, Min support: 15, Min complexity filter: 0.3, LCA algorithm: weighted) [49].

### Statistical analysis of read – based metagenomics data

**Figure 2.**
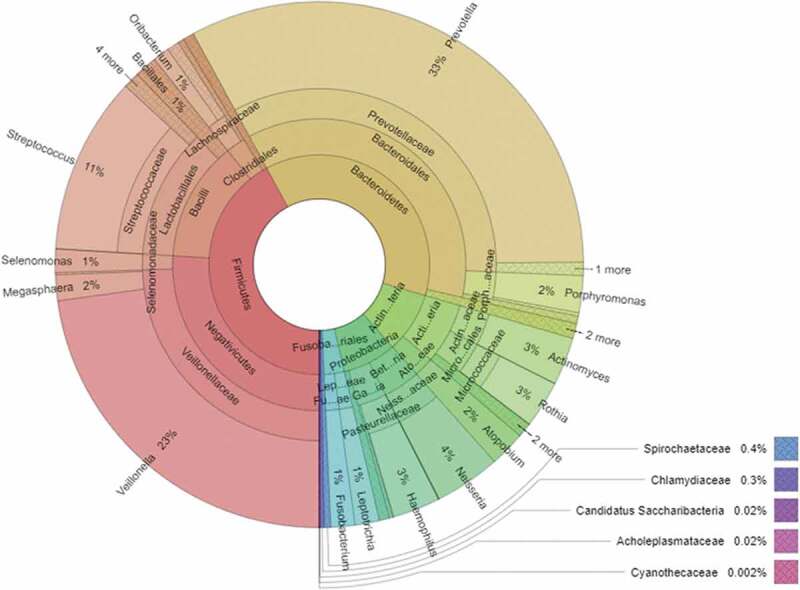
Overall composition of the salivary microbiome of study participants including the metagenomes of both smokers and non-smokers. Due to space limitations, only the most relevant bacterial genera, families, orders and phyla are indicated; the bacterial classes are not shown.

**Figure 3.**
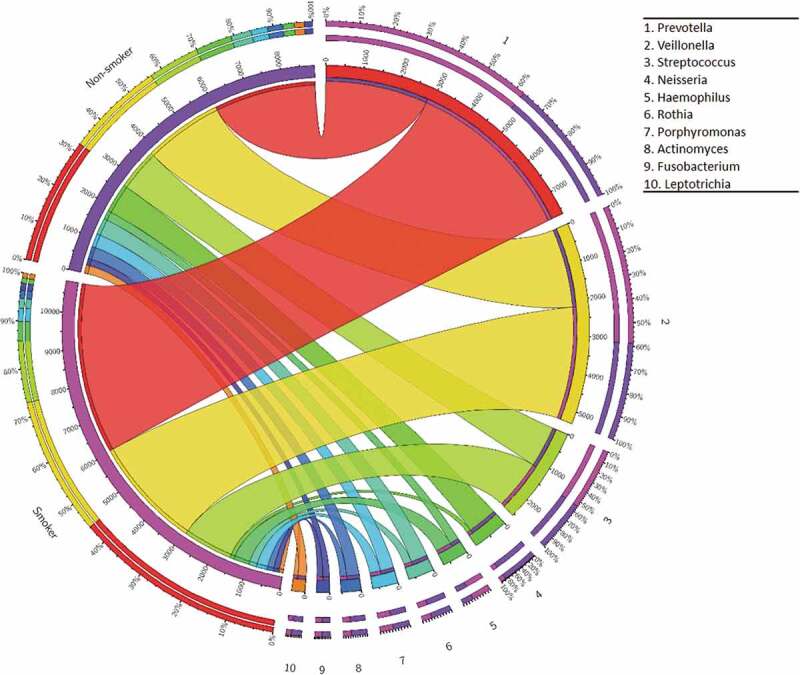
Relative abundance of the 10 most abundant bacterial genera in non-smokers and current smokers. Circos plot illustrating the most abundant bacterial genera listed in inset from 1 to 10. The widths of the bands are proportional to the abundance of the particular taxon in the two study groups.

**Figure 4.**
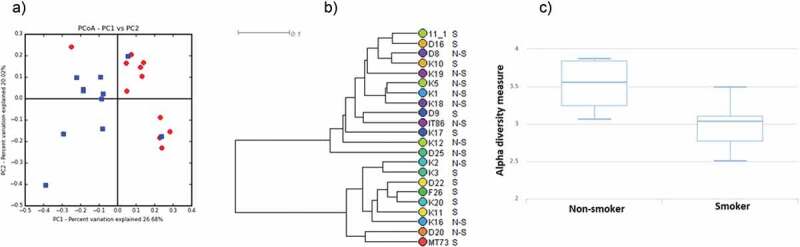
Diversity of the salivary microbiome in non-smokers and current smokers.

MEGAN6 was used to investigate microbial communities and export data for statistic calculations. UPGMA (Unweighted Pair-Group Method with Arithmetic Mean) with Bray–Curtis method was employed to cluster the samples (Figure 4(b)). Rarefaction estimation was performed by MEGAN6 [49] (Supplementary figure 1). Krona program was used to visualize the average composition of microbial taxa [50] (Figure 2). The distribution of top 10 most abundant microbes between the two sets of samples was presented with Circos [51] (Figure 3).

For microbial core and diversity calculation MetaCoMET (Metagenomics Core Microbiome Exploration Tool), an interactive web tool, was used. Shannon statistical method was performed to calculate alpha diversity (Figure 4(c)). Emperor program (integrated into MetaCoMET) carried out the principal component analysis (Figure 4(a)). For core calculation default parameter sets were fixed with the persistence Venn diagram type [52].

(a) Principal component analysis of salivary microbiomes of non-smokers and current smokers. Symbols: blue squares = non-smokers; red diamonds = current smokers (b) Hierarchical clustering analysis of salivary microbiomes of non-smokers and current smokers by UPGMA (unweighted pair group method with arithmetic mean). The two metagenome clusters characterizing the two very distinct study groups are boxed. Comparison of Shannon diversity indices. The alpha diversities are clearly distinct although statistically not rigorously different.

**Figure 5.**
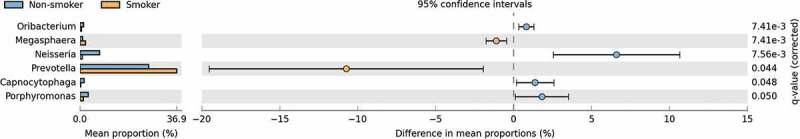
Bacterial genera which differ significantly in relative abundance between the salivary samples of non-smokers and current smokers. The statistical analysis was performed and visualized using the STAMP package. Mean abundance (mean proportion) and difference in mean proportion for genera showing significant difference in abundance are shown. The 95% confidence intervals and statistical significance (corrected **q** value) are indicated as well.

Statistical Analysis of Metagenomic Profiles (STAMP) was used to compute the abundance differences in the case of whole microbiome. Dissimilar taxa were identified with two-sided t-test at 0.95 confidence intervals and the results with q-value (corrected p-value) of <0.05 were retained. In STAMP minimum difference between proportions was set to 0.3 and Storey FDR (False Discovery Rate) filtered out false positive significant differences [53,54] (Figure 5).

### Genome-based evaluation of the sequencing data

**Figure 6.**
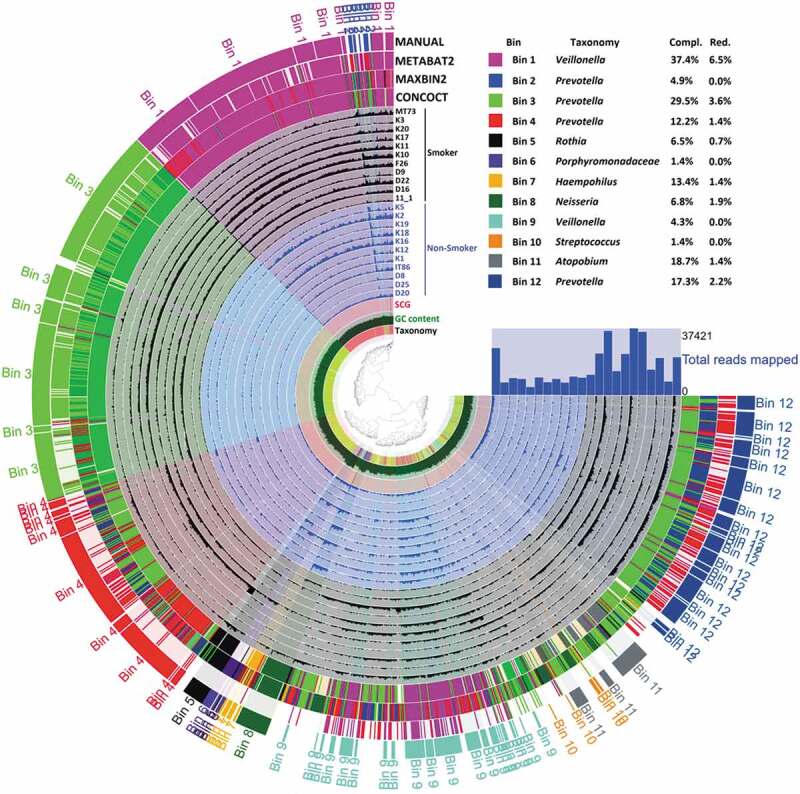
Analysis of shotgun sequences by genome-centric binning. Distribution of contigs built from filtered sequences of salivary bacterial communities. The grouping of contigs based on sequence-assignments of automated binning programs METABAT2, MAXBIN2 and CONCOCT as well as manually defined bins were visualised by the Anvi’o platform. SCG: single-copyy genes; GC: guanine-cytosine (GC) content.

Filtered sequences produced by Prinseq were co-assembled with Megahit (Minimum contig length: 1000 bp, Minimum k-mer size: 21, Maximum k-mer size 141) [55]. After simplifying the header of contig FASTA file using the Anvi’o script, Bowtie2 was employed to map back the original sequences to the contigs [56]. Then, we used Anvi’o V5 to follow the ‘metagenomics’ workflow [57]. Briefly, contig database was generated in the first step, where open reading frames were identified by Prodigal and contig k-mer frequencies were computed. Then, Hidden Markov Modell (HMM) of single-copy genes was aligned by HMMER [58–61]. We used InterProScan v5.31–70 and the metagenome classifier Kaiju to functional and taxonomic annotation of contigs [62–65]. The outputs were imported into the contig database. BAM files, made by Bowtie2, were used for profiling the contig database, this way we generated sample-specific information about the contigs (i.e. mean coverage). These were merged together. Three automated binning programs, i.e. CONCOCT, METABAT2 and MAXBIN2, were employed to reconstruct microbial genomes from the contigs (Minimum length: 2,000 bp) [66–68]. We also used the Anvi’o human-guided binning and ‘anvi-refine’ options [57]. The binning results were incorporated to the contig database. Anvi’o interactive interface was employed to visualize and summarize the data in Figure 6.

## Results

### Lifetime tobacco exposure in current smokers

Lifetime tobacco exposure of current smokers was calculated in pack-years by multiplying the average number of packs of cigarettes smoked per day by the number of years the person had smoked. The mean smoking (tobacco) exposure of current smokers (N = 11) was 11.5 ± 8.2 pack-years (range: 0.6–23 pack-years) (Supplementary table 1).

The distribution of early periodontal lesions (mild gingivitis) was equal in both groups. The number of decayed teeth was higher in the group of smokers (4.18 SD: 0.78) compared to the group of non-smokers (2.45 SD: 0.85) but the difference between the group was not significant (P < 0.1499 Student’s test).

### Exhaled carbon monoxide in healthy smokers and non-smokers

Data regarding exhaled CO levels were available for all non-smokers (N = 11; mean exhaled CO level: 1.7 ± 0.9 ppm) and for 6 of the 11 current smokers (mean exhaled CO level: 12.3 ± 12 ppm). The difference between the exhaled CO level of non-smokers and current smokers was statistically significant (Studen’s t-test; P˂0.0001). The values found in our study were comparable to the data recorded for healthy adult non-smokers (1.5 ± 0.6 ppm) and healthy adult smokers (9.7 ± 5.7 ppm) in a recent independent study using the same type of CO monitor [69].

### Read-based characterization of the salivary microbiome in non-smokers and current smokers

Shotgun metagenome sequencing of salivary DNA samples from 22 individuals (11 non-smokers and 11 current smokers) resulted in 256,567 quality-filtered reads; mean reads per sample: 11,662.1 (SD: 2,261.95). To monitor the efficiency of sequencing, rarefaction curves were computed (Supplementary figure 1). The rarefaction curves reached their asymptotes around 5,000 reads, indicating that the sequencing depth was sufficient to cover almost all genera in the bacterial communities analyzed.

Based on the alignment of the quality-filtered reads with sequences in the NCBI nr database, 66 bacterial genera could be identified. The overall composition of the oral microbiome of study participants (both non-smokers and current smokers) is shown in Figure 2. It is apparent that three genera, i.e. *Prevotella, Veilonella and Streptococcus*, predominated in the saliva of the Hungarian study participants, although the mean relative abundance of 11 additional genera reached or exceeded 1% (Supplementary table 2).

Comparison of the salivary microbiome of non-smokers and current smokers revealed that 48 bacterial genera were present in at least one specimen of both groups (Supplementary table 2). All of the 14 abundant genera (relative abundance 1% or higher) were shared by both microbiota. In addition, 34 rare genera (relative abundance ˂1%) were also shared by non-smokers and current smokers. In total, 8 rare genera were unique to non-smokers whereas 10 rare genera manifested themselves in current smokers only (Supplementary table 2).

The relative distribution of the 10 most abundant genera is presented in Figure 3. It is noteworthy that the genera *Prevotella, Veillonella* and *Streptococcus* predominated in the saliva samples of current smokers, comprising about 90% of the total reads. In the non-smoker group, about 70% of all reads mapped to these 3 genera. In accordance with PCA (principal component analysis) (Figure 4(a)), hierarchical clustering analysis by UPGMA (unweighted pair group method with arithmetic mean) (Figure 4(b)) also showed that the microbiomes of non-smokers and current smokers did not form two rigorously separated clusters although the tendencies with a few outliers are clearly recognizable. We also noticed that a group of non-smokerss (K19, K5, K1, K18) and a group of current smokers (K3, D22, F26, K20, K11) were clearly located on two separate branches of the UPGMA tree (boxed in Figure 4(b)). This might be a sign of distinction albeit statistically non-significant. The Shannon index, which reflects both richness and evenness of microbial communities, was higher in case of the salivary microbiome of non-smokers, compared to that of the current smokers (Figure 4(c)). The difference, however, was again not statistically significant.

In spite of thee similarities in the composition and diversity of salivary microbial communities, the relative abundance of distinct genera differed significantly between non-smokers and current smokers. Two genera displayed pronounced changes. *Prevotella* (order *Bacteroidales*), the predominant genus in both groups, was more abundant in the saliva of current smokers (mean relative abundance: 36.8 ± 9.8%), compared to non-smokers (mean relative abundance: 26.0 ± 9.9%); the difference between the groups was statistically significant (p = 0.044; Figure 5). In addition to *Prevotella*, the genus *Megasphaera*, a member of the order *Negativicutes*, belonging in the phylum *Firmicutes*, was also enriched in the saliva samples of current smokers (Figure 5). Although *Megasphaera* was a rare, low-abundance genus in the salivary bacterial communities of non-smokers, it reached a relative abundance above 1% in the salivary samples of current smokers. In contrast, the salivary microbiome of non-smokers was significantly enriched in the genera *Neisseria* (phylum: *Proteobacteria*; order: *Neisseriales*), *Oribacterium* (phylum: *Firmicutes*; order: *Clostridiales*), *Capnocytophaga* (phylum: *Bacteriodetes*; order: *Flavobacteriales*) and *Porphyromonas* (phylum: *Bacteriodetes*; order: *Bacteriodales*) (Figure 5).

### Analysis of shotgun sequences by genome-centric binning

**Figure 7.**
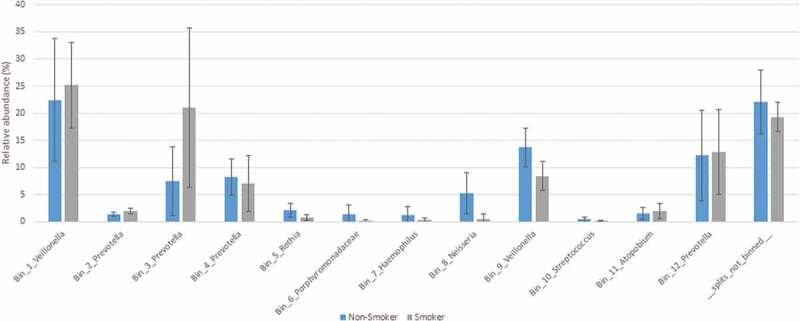
Relative abundance of bacterial genera identified by genome-centric binning. *Prevotella* (bin 3) showed a significantly higher relative abundance in the salivary microbiome of current smokers, whereas the family *Porphyromonadaceae* (bin 6) and the genus *Neisseria* (bin 8) were significantly more abundant in the salivary microbiome of non-smokers.

In addition to read-based taxonomic classification, the filtered sequences generated in our study were separately assembled in contigs, which were clustered into bins based on their inherent sequence features (genome-centric binning). The workflow of both read-based classification and metagenomics binning is presented in Figure 1. A total of 12 bins were constructed, using the human-guided automated binning programs that rely on co-abundance of sequences as well as compositional information such as GC content, tetranucleotide frequencies and identification of single-copy genes (Figure 6). The putative genomes of the genera *Prevotella* and *Veillonella* each were represented by 4 and 2 separate bins, respectively, whereas the genera *Rothia, Haemophilus, Neisseria, Streptococcus* and *Atopobium*, as well as the family *Porphyromonadaceae* each occupied a single distinct bin. Based on genome-centric binning, *Prevotella* (bin 3) showed a significantly higher relative abundance in the salivary microbiome of current smokers, whereas the family *Porphyromonadaceae* (bin 6) and the genus *Neisseria* (bin 8) was significantly more abundant in the salivary microbiome of non-smokers (Figure 7). These data are compatible with the results of read-based taxonomic classification of salivary microbiomes from non-smokers and current smokers (see Figures 3 and 5) and therefore the two distinct metagenomic approaches mutually validate each other.

## Discussion

Toxic and mutagenic chemicals as well as particulate matter in cigarette smoke may initiate oral diseases either *via* direct interactions with human cells in the oral cavity, or indirectly, by affecting the environment of these cells such as the extracellular matrix and/or the oral microbiome [12,25,32,70,71]. Mutagens may cause genetic damage both in human cells and in bacterial cells inhabiting the oral cavity [31,70]. In addition, smoking may damage oral microcirculation and create an acidic, relatively hypoxic milieu in the oral cavity that may favour the growth of distinct members of the oral microbial community [72,73]. Establishment of a similar microenvironment may select for the local growth of distinct anaerobic bacteria within neoplastic tissues, too [74,75].

Saliva is a complex biological fluid bathing the various anatomical structures of the oral cavity covered by biofilm-forming microbial communities. From mature biofilms located at intraoral surfaces, bacteria – including potential pathogens – are regularly shed into the saliva. In such a planktonic state, oral bacteria may be transmitted to new ecological niches [13,76].

Oral diseases and tobacco smoke exposure induce anatomical and physiological changes in the oral cavity, consequently the composition of surface-attached bacterial supragingival and subgingival biofilms and the structure of the salivary microbial community may be altered [17,77–81]. Smoking cessation may revert, however, the changes in the composition of oral microbiome [18,82]. Our study groups of current smokers and non-smokers comprised middle-agedd female and male healthy Hungarian citizens, having a cigarette smoking habit of various degrees. The tobacco consumption and the duration and extent of tobacco smoke exposure varied considerably (Supplementary table 1). The justification for selecting such a diverse group of individuals was that we attempted to dissect a broad sample covering diverse individual variability in the environmental and lifestyle parameters of our subjects in order to track the general features of oral microbiome alterations caused by cigarette smoking. As expected, the price for this heterogenous sampling approach was substantial individual deviation in microbial communities within the two study groups. In spite of the dissimilarities in their individual case histories, the “current smokers” and “non-smokers” demonstrated clear difference in their exhaled CO levels, a good indication of the substantial physiological influence caused by deleterious cigarette smoking regardless of other variables. The diversity of individual microbes affected by environmental and lifestyle conditions other than smoking habits masked several aspects of oral microbiome rearrangements as being statistically not significant although indicating noticeable trends (Figure 4(a,b)). Accordingly, the difference was not rigorously significant, we found that the Shannon index which reflects both richness and evenness of microbial communities, was higher for the salivary microbiome of non-smokers compared to current smokers (Figure 4(c)). This may be related to a decreased evenness in the microbial community of current smokers which is characterized by an increased abundance of *Prevotella* (Figures 3 and 5). Similarly, the microbiomes of non-smokers and current smokers did not form well separated clusters by principal component analysis (PCA) and hierarchical clustering (UPGMA). Nevertheless, location of a group of samples from non-smokers and another from current smokers on two separate branches of the UPGMA tree might be a sign of distinction between the two microbiomes (Figure 4(a,b)).

In the thorough shotgun full metagenomic study we found that all of the 14 abundant genera (relative abundance 1% or higher) and 34 rare genera (relative abundance ˂1%) were shared by the salivary microbiomes of non-smokers and current smokers, whereas 8 rare genera were unique to non-smokers and 10 rare genera were unique to current smokers (Supplementary table 2). Remarkably, the read-based metagenomics data were supported by metagenomic binning sufficiently (Figures 6 and 7).

Both microbial communities were predominated by 3 genera, i.e. *Prevotella, Veillonella* and *Streptococcus* (Figure 3). Analysis of the complexity of salivary microbial communities in non-smokers and smokers gave variable results in previous studies. In most cases, analysis of oral wash samples or oral swabs did not reveal significant differences between the microbiomes of smokers and non-smokers [83–85]. To the contrary, a large study of oral wash samples revealed a significant difference in overall oral microbiome composition between current and non-current (former and never) smokers [18]. Further studies may resolve these apparent discrepancies.

We observed that although the overall composition of the oral microbiome did not differ substantially between non-smokers and current smokers (Figures 3 and 4) the relative abundance of two distinct genera, *Prevotella* and *Megasphaera*, was higher in salivary samples of current smokers (Figure 5). *Prevotella* species are Gram-negative, anaerobic bacteria which belong in the phylum *Bacteriodetes*. Although in healthy humans the *Prevotella* genus is one of the dominant genera of the salivary microbiome, distinct members of the genus *Prevotella* are associated with inflammatory diseases and may facilitate carcinogenesis as well [86-88, reviewed by 89,90]. There are, however, contradicting observations regarding the role of *Prevotella* in the development of oral cancer [14,74,79,91–94]. Thus, it remains to be established whether members of the genus *Prevotella* are opportunistic inhabitants of malignant tumors or play a causative role in oral or colorectal carcinogenesis [95,96].

*Megasphaera*, the other genus of increased abundance in the saliva of current smokers belongs in the phylum *Firmicutes. Megasphaera* are Gram-negative anaerobic cocci, which reside in the upper digestive tract of adults, contributing to the microbial community of tongue dorsum, tonsils and saliva [86]. In line with our results, an increase in relative abundance of *Megasphaera* was observed in oropharyngeal samples and esophageal samples of smokers relative to non-smokers [97,98]. Perhaps the smoky environment may confer a growth advantage for *Megasphaera* [98]. Dysbiotic diseases, including periodontitis and bacterial vaginosis were also associated with higher relative abundance of *Megasphaera* species [99,100]. Moreover, the genus *Megasphaera* was associated with human papillomavirus (HPV) positive head and neck squamous carcinoma and lung cancer [101,102].

Our finding of an increased abundance of *Prevotella* and *Megasphaera* in the saliva of current smokers may facilitate the initiation and progression of various pathogenic processes within the oral cavity and may affect the composition of microbial communities along the route of swallowed saliva as well, i.e. on the surface of tonsils and throat, and possibly even the esophageal mucosa [86,98].

We observed that the salivary microbiome of current smokers was significantly depleted relative to non-smokers in the genera *Neisseria* (phylum: *Proteobacteria*; order: *Neisseriales*), *Oribacterium* (phylum: *Firmicutes*; order: *Clostridiales*), *Capnocytophaga* (phylum: *Bacteriodetes*; order: *Flavobacteriales*) and *Porphyromonas* (phylum: *Bacteriodetes*; order: *Bacteriodales*) (Figure 5). The decreased abundance of *Neisseria* in the saliva of current smokers could possibly be attributed to the selective toxicity of cigarette smoke for *Neisseria* species [103,104]. Compared to non-smokers, the abundance of *Neisseria* decreased in the oropharynx of smokers [97].

Increased abundance of *Oribacterium parvum* and other distinct bacterial species in prediagnostic oral wash samples was associated with a decreased risk for esophageal adenocarcinoma development [15]. An increased level of *Oribacterium* was also detected in oral rinse samples of patients with oral cavity carcinoma and oropharyngeal carcinoma [14,101]. Therefore, it is not easy to predict how the decreased salivary level of *Oribacterium*, observed in our current smoker study group, may affect pathological processes in the gastrointestinal tract.

Analysis of relative abundance of *Capnocytophaga* species in the oral cavity of smokers and non-smokers also yielded apparently conflicting results in various laboratories. Thomas et al. observed an increased level of *Capnocytophaga* in oral swab samples of smokers, whereas Wu et al. found a decreased level in oral wash samples of smokers [18,84]. Our finding, based on saliva samples collected by the simple drooling method, is in accordance with the data of Wu et al. [18]. One may assume that using oral swab samples may permit a more efficient collection of bacteria deeply embedded in biofilms at various surfaces compared to taking unstimulated saliva samples or oral wash samples.

Nicotine, an important component of cigarette smoke, inhibited the growth of *Porphyromonas gingivalis* [105]. This observation may explain the decreased level of *Porphyromonas* species in saliva samples of current smokers, compared to non-smokers, as demonstrated in our current study, in accordance with previous findings [18,83].

In summary, we conclude that probably several environmental, personal health history and life style factors affect the alterations in the oral microbiota in smoker and non-smoker individuals. Our study did not address most of these potential factors, which may blur somewhat the picture. Nevertheless, the clear message of this study is that tobacco smoking brings about dysbiotic, deleterious changes in the oral microbiota and this may lead to severe oral and general unwanted health consequences for the smoking people. We also identified a few genera for diagnostic purposes of the warning molecular taxonomy signs of the dangerous consequences for smokers.

## Supplementary Material

Supplemental Material
