# Supplementary figures and images for: A case study of salivary microbiome in smokers and non-smokers in Hungary: analysis by shotgun metagenome sequencing
Source: J Oral Microbiol. 2020 Jun 7;12(1):1773067. doi: 10.1080/20002297.2020.1773067 (PMC7448927; doi:10.1080/20002297.2020.1773067)

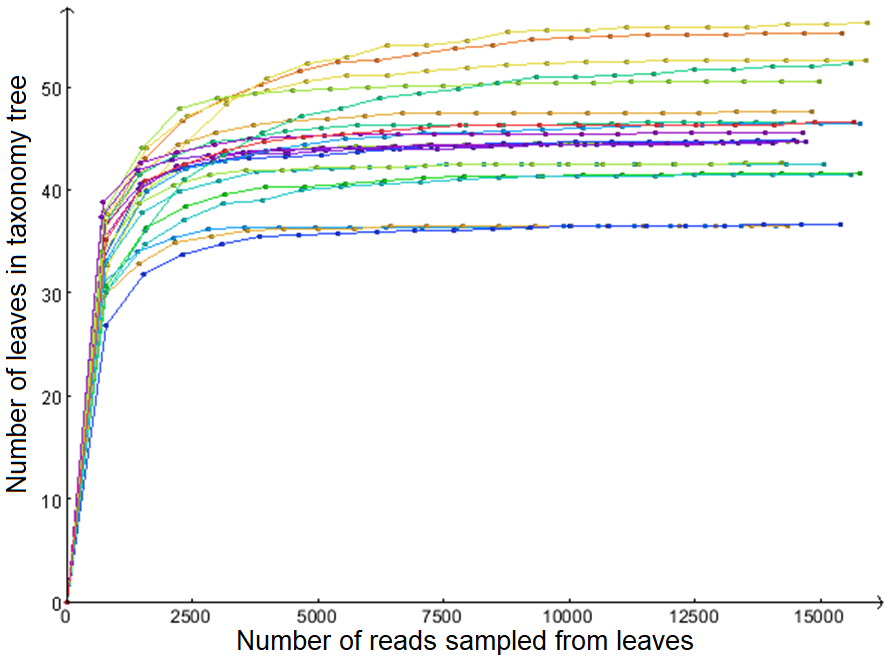

Supplement: Supplemental Material [file ZJOM_A_1773067_SM6668.tif]
